# Biophysical Enhancement in Fracture Healing: A Review of the Literature

**DOI:** 10.7759/cureus.37704

**Published:** 2023-04-17

**Authors:** Ioannis D Aifantis, Margarita-Michaela Ampadiotaki, Dimitrios Pallis, Konstantinos K Tsivelekas, Stamatios A Papadakis, Efstathios Chronopoulos

**Affiliations:** 1 2nd Orthopedic Department, KAT Attica General Hospital, Athens, GRC; 2 Orthopedic Surgery Department, Konstantopoulio General Hospital, Athens, GRC

**Keywords:** extracorporeal shockwave therapy, electrical stimulation, laser, ultrasound, electromagnetic fields

## Abstract

Bone healing constitutes a complex process involving cellular and pathophysiological mechanisms. Despite progress in osteosynthesis techniques, fracture union continues to be challenging. In some cases, it is not ultimately achieved or is delayed relative to the expected time resulting in economic and social outcomes for the patient and the health system. In addition to surgical treatment, biophysical methods have been developed to assist in fracture healing used in combination or individually. Biophysical stimulation is a non-invasive therapy used in orthopedic practice to increase and enhance tissue's reparative and anabolic activities. This study reviewed the existing literature, including electromagnetic fields, ultrasound, laser, extracorporeal shockwave therapy, and electrical stimulation, and revealed the efficacy of biophysical stimulation for bone healing. This study aims to define if these methods are helpful, especially in cases of non-union. Biophysical stimulation requires care and precision in use to ensure the success expected of it by physicians and patients.

## Introduction and background

As the population aging increases, the incidence of fractures increases, especially in elderly patients. Also, fractures in younger patients have increased due to high-energy trauma, such as motor vehicle accidents. The rate of complications remains high, and more precisely, the percentage of nonunion (range 5-10% in the USΑ), that is, the failure to achieve union [1]. This entails an increase in hospitalization, morbidity, and mortality in elderly patients and costs for both the health system and the patient resulting in social, psychological, and economic consequences [2,3]. In cases of delayed union and nonunion, revision of osteosynthesis is mandatory, requiring the use of bone grafts which prolongs the patient's morbidity. A handful of means have been described to find non-invasive biophysical methods to enhance porosity, even in cases with an increased risk of nonunion. The surgeon should be aware of the pathophysiological mechanism of bone healing, the specific characteristics of the fracture, and the patient's medical history [4].

## Review

Materials and methods

This study aims to review the contribution of biophysical methods to bone healing. The methods that are studied in the databases were "electrical stimulation," "extracorporeal shockwave therapy," "electromagnetic fields," low-intensity pulsed "ultrasound," and "laser" therapy. In order to achieve the aim of this study, the literature was reviewed in the following databases: PubMed, Cochrane, Scopus, Google Scholar, and Embase. Articles in English and available in full form were included. No restriction was placed on the date of publishing. Finally, all published studies that met the following criteria were included: English language, human studies, and adult patients. 

Electrical Stimulation 

Stimulation by a direct electric current is the first mentioned method of fracture healing enhancement. Its application is based on the fact that the diaphysis has a more positive electric charge than epiphysis and metaphysis, and the fracture site is negatively charged. The electric current in this area activates osteoblastic and osteoclast activity [5]. In experimental studies, there has been an increase in mitotic activity, local aggregation, and differentiation in the progenitor forms of osteocytes and an improvement in the rate of intercellular intervention. Although the progress of porosity using electricity has been investigated in several clinical studies over the past 30 years, it is not widely used in clinical practice [6]. Micro-electrical fields are currents with a voltage of less than 1 mA. They can be continuous or alternating. The currents preferred in nonunion cases are continuous with a low voltage and the negative pole placed on the fracture. They induce the proliferation of cells and collagen. Microcurrents do not produce muscle contraction. Animal studies have shown that the electric current when applied to the fracture, induced osteogenesis through primary and secondary healing [5]. A review published in 2011 reported non-significant differences for electrical stimulation in improving nonunion rates in four trials involving 125 patients [7]. Also, a survey of 450 Canadian trauma surgeons in 2008 showed that 23% of the surgeons used bone electric stimulators to accelerate bone healing [8]. In the literature, the case series provided 69% and 73% healing rates after capacitive coupling (CC): treatment during 30 and 26 weeks, respectively. In accordance with the aforementioned studies, Kleczynski S, in a series of 34 patients, recorded an 88% rate of tibia bone healing. However, the healing time averaged over 10 months [9]. Ahl T et al. [10] found a significantly lower percentage (43%) of porosity and attributed seven of the 13 failures to the mobility of the descending limbs. Negative potentials are created by compression, and positive ones by voltage. Within this frame of reference, the bone is formed by negative potentials and absorbed by positives. Electrical potentials are generally formed in response to charge [11,12]. The application of electrical irritation to nonunion was first described by Brighton CT et al. in 22 patients [13]. They stated that the rate of healing was 77% over 27 weeks and reported local rash from the electrode as a complication. This theory introduced the application of electric fields to caterpillars in order to achieve healing. Electrical excitation is achieved in three ways: continuous electric current, capacitive and inductive coupling. Contraindications to their use are bone deficit, congenital false articulation, mechanical instability, and infection [13].

Direct electrical stimulation (DC): This is an invasive technique that involves the implantation of an electrode in the bone. Specifically, the anode electrode is placed in the area centered on the fracture. The following reaction reduces the concentration of oxygen, increasing pH and hydrogen peroxide. Also, the direct electric current increases proteoglycans and collagen. Hydrogen peroxide stimulates macrophages that release vascular endothelial growth factor (VEGF), which affects osteogenesis [14,15]. Capacitive coupling (CC): It is a non-invasive technique that involves the placement of two electrodes on either side of the fracture and using alternating current. The proliferation of osteocytes is accompanied by an increase in the intracellular concentration of calcium as it transfers them into the cell. In many studies, it has been shown that signal transduction results from the translocation of calcium ions through channels that, in turn, lead to an increase in prostaglandin and calmodulin. In addition, capacitive coupling increases bone morphogenetic proteins (BMPs) and Noggin [16]. In the literature, many studies do not suggest a significant benefit of using electrical stimulation in fracture healing [17-24]. However, the studies are characterized by a large heterogeneity of patient characteristics and fracture selection. On the other hand, electrical irritation seems to have an effect on reducing pain. Randomized studies are needed to reach more robust conclusions (Table 1).

**Table 1:** Electrical stimulation.

Author	Patients	Union rate (%)
Abeed RI et al. [17]	16	69
Brighton CT et al. [13]	22	77
Zamora-Navas P et al. [18]	22	73
Ahl T et al. [10]	23	43
Brighton CT et al. [16]	178	84
Kleczynski S [9]	34	88
Bassett CA et al [19]	24	71
Bassett CA et al. [20]	46	87
Bassett CA et al. [20]	83	90
de Haas WG et al. [21]	17	71
de Haas WG et al. [22]	44	84
Dunn AW et al. [23]	31	81
Heckman JD et al. [24]	149	64

Low-Intensity Pulsed Ultrasound (LIPUS)

In the literature, LIPUS accelerated fracture healing is controversial, with many controlled studies showing a positive effect [25]. Ultrasound produces frequencies that are not perceived by humans, greater than 20 kHz, and is used for imaging tests, physiotherapy, and during surgeries. LIPUS is used to achieve fracture healing [26, 27]. The first description of LIPUS was by Maitz G in 1950 to laboratory animals, who suggested that at high frequencies, it causes a thermal burn, but in smaller ones, it promotes healing [28]. The FDA accepted the use of ultrasound in treating fractures in 1994 and the treatment of nonunions in 2000 [25,29]. The mechanism of action of LIPUS has been studied by many authors. A study by the University of Bristol showed that the healing time in healthy laboratory animals and experimental animals with diabetes was the same by its use. Its mechanism of action seems to involve the activation of integrins in ultrasound-stimulated cells [30, 31]. Another theory bases its action on the nanomotor it produces. It thus triggers a signaling cascade by activating the integrin receptors and thus increasing the BMPs, transforming growth factor-β (TGF-β), VEGF, and vascularity in the fracture area. It has been proven that the application of ultrasound increases ionic conductivity by up to 22% and reduces the need for ATP consumption [32,33]. In the literature, a study by Giannoudis PV et al. introduced the term "diamond concept" and defined four factors that predict porosity. If there is a fracture gap >10 mm, then revision surgery is necessary, and the use of ultrasound is not sufficient [34]. The other two factors of the "diamond concept" concern growth factors and osteogenetic capacity, which depends on the patient's biology. In this case, ultrasound is useful to facilitate healing. Although, there are studies using ultrasound immediately postoperatively with a healing rate of 96.5% [35, 36]. Other studies used ultrasound 12 months after the fracture with a healing rate of 86.2 % [36]. Noteworthy to mention is the result of the study of Jingushi S et al., who concluded that the absence of radiological fracture healing progress after four months of ultrasound indicated the revision surgery, and the probability of eventual union being reached, is estimated at 92% (Table 2) [37].

**Table 2:** Low-intensity pulsed ultrasound.

Author	Patients	Union rate (%)
Nolte PA et al. [38]	28	86%
Mayr E et al.[39]	100	86%
Lerner A et al.[40]	17	93.3%
Pigozzi F et al.[41]	15	100%
Gebauer D et al. [42]	66	85%
Jingushi S et al. [37]	72	75%
Rutten S et al.[43]	71	73.2%
Hemery X et al. [44]	14	78.5%
Schofer MD et al. [45]	51	64,7%
Roussignol X et al. [46]	60	88,1%
Watanabe Y et al. [47]	151	72,1%
Zura R et al. [36]	767	85,7%

The literature review suggests that using LIPUS for nonunion leads to an increased union rate, especially when applied within three to six months of the last surgery. Hypertrophic nonunion seem to be more favored than atrophic pseudoarthrosis. Several of the above studies used LIPUS as an adjunct to surgery. Initial treatment was conservative in eight and surgical in 21 studies, respectively. To our knowledge, recent data in the literature demonstrate a positive effect on fracture healing, but randomized studies are essential to point out the beneficial effect [38-47]. Also, the use of ultrasound seems to be useful in patients who due to serious perioperative risk cannot undergo surgery [48].

Pulsed Electromagnetic Field (PEMF)

The first description of the application of electrical stimulation was in 1841. However, its use was not widespread until 1950, when an experimental study was carried out on rabbits that were electrically stimulated, and new bone was produced [48]. The most widespread PEMF devices use non-invasive induction coupling and enable application by any surgical method. Electric current is generated by a coil and stimulates the action of osteoblasts as it increases the perfusion and calcification of bones. The magnetic fields used are from 0.1 to 20 G and produce electric fields of 1-100 mV/cm [49]. Many authors suggested that electromagnetic fields act by a mechanism similar to that of mechanical charge [50]. Electromagnetic fields increase the production of proteins in the extracellular matrix and transcription factors [50]. They increase cell membrane permeability and affect membrane receptors such as parathyroid hormone (PTH), IGF-2, LDL, calcitonin and insulin, and morphogenetic proteins. Also, they achieve osteocyte proliferation and production of alkaline phosphatase, runx2, Nf-kB, and metalloproteins. They react directly on progenitor mesenchymal cells and promote their differentiation as they increase bone density and interleukin-6. Another possible mechanism of action of electromagnetic fields is that they increase the levels of angiopoietin II and intraluminal perfusion [51]. Contraindications to using magnetic fields are infection, bone deficit, and insufficient fracture stabilization. Most studies recommend using magnetic fields after diagnosing pseudoarthrosis or delayed union, while few studies suggest their use immediately postoperatively [49]. The first study in the electromagnetic fields was conducted in 1980 by de Haas WG et al. and included 17 patients with a union rate of 88.2%. Despite the small sample of patients, this study is decisive because it is the first to highlight the beneficial effects of electromagnetic fields [21]. On the contrary, Bassett CA et al. stated that their use is based on the theory of piezoelectricity of collagen, and the applied voltage regenerates magnetic stresses along its fibers. The electromagnetic radiation used in these cases has a long wavelength and frequency compared to other methods and is applied pulsed, with an on-off periods sequence. Also, it does not produce heat and thus does not become harmful to tissues [20]. Human tissues are polarized, and the exchange of substances through the cell membrane depends on the ions on either side. PEMFs affect polarity and therefore affect the exchange of substances through membranes and trigger differentiation and protein synthesis [52-55]. Recent studies have shown that electromagnetic fields have beneficial effects as they accelerate the time to healing of fractures [54-65]. A study by Bassett CA reported 127 tibia nonunions using PEMF for 5.2 months, with an 87% union rate [63]. Sharrard WJ stated 86.7% of porosity rates with magnetic fields while pointing out that the bone failure was due to infection, dissociation, and inadequate immobilization of fractures [56]. Magnetic fields release endorphins, act as analgesics, regulate fluid exchange, reduce edema, and increase WBCs, gamma-globulin, and blood supply (Table 3).

**Table 3:** Pulsed electromagnetic field.

Author	Patients	Bone	Union rate
Mammi GI et al. [55]	37	Tibial osteotomies	72.2%
Sharrard WJ[56]	51	Tibia	86.7%
Simonis RB et al. [57]	15	Tibia	13/15
Faldini C et al. [58]	32	Subcapital femoral fracture	94%
Hannemann PF et al. [59]	53	Scaphoid	No significant difference between placebo group and control group
Shi HF et al. [60]	58	Long bones	77.4%/48.1% (Control/Placebo)
Barker AT et al. [62]	17	Tibia	55.6%
Scott G et al.[61]	23	Tibia	60%
Bassett CA[63]	127	Tibia	87%
Assiotis A et al. [64]	44	Tibia	77.3%
Martinez-Rondanelli A et al.[65]	63	Femur	94%/87% (Control/Placebo)
Streit A et al. [66]	8	Fifth metatarsal	100%

Contraindications to its use are pacemakers, malignancy, pregnancy, and implants [67-68]. However, porosity depends on other local and systemic factors mentioned earlier, which must be considered. More studies are needed to eliminate heterogeneity between patients in order to publish more secure conclusions [68]. 

Low-Level Laser Therapy (LLLT)

The laser reacts as an amplification of light through forced emitting radiation. The laser stimulates biochemical changes at the cellular level and functions like photosynthesis in plants, where photons are absorbed by cellular photoreceptors. These devices produce an electromagnetic wave with monochromaticity and consistency [69]. Low-level laser has been used in physiotherapy (10 mW-500 mW). Laser with a wavelength in the red to the infrared region is used in these cases because it has the ability to penetrate the skin and tissues. The power density is usually 5W/cm2 and is applied for 30-60 seconds per time. Inflammation reduction, pain relief, and tissue regeneration are achieved with its use. The laser at low doses has been shown to enhance the cellular proliferation of fibroblasts, keratin cells, and lymphocytes [69]. The mechanism is the photostimulation of mitochondria that eventually leads to the proliferation of growth factors and enhances angiogenesis and collagen synthesis. Also, it has been shown to present a two-phase curve where lower doses are more effective than higher doses. According to the guidelines, a laser should not come into contact with the eyes and should not be applied to an area with a neoplasm during pregnancy or to epileptic patients [70]. With the use of the laser, there is an increase in certain genes that stimulate both the proliferation and differentiation of osteoblasts, eventually forming a newly formed bone. In general, clinical effects on the human body include the following: reduction of inflammation and pain, faster healing of skin, tendons, and muscle, acceleration of fracture healing, stimulation of angiogenesis, bacteriostatic and vasodilator action [69]. Most studies in the literature involved laboratory animals, but in our study, only articles referring to humans have been referred to. A study included 50 patients suffering from wrist fractures who were treated conservatively and divided into two groups, pointing out that half of the patients underwent a laser treatment program five times a week for two weeks, and the remaining participants underwent a placebo. Pain, functional effect, and grip strength were evaluated before and after treatment. After treatment, the laser group showed significant changes in all parameters. After treatment and during monitoring, the comparison of those groups showed significant differences in all parameters. LLLT can relieve pain and improve the procedure for healing wrist fractures [71].

Extracorporeal Shockwave Therapy (ESWT)

Ultrasound has been widely used in the treatment of nephrolithiasis. Haupt G et al. in 1991 observed that patients who had undergone shock ultrasound for nephrolithiasis had hypertrophy of the iliac bone, but the mechanism was unknown [72]. Recent animal studies have demonstrated that the impact of ultrasound promotes the production of porous. Also, ultrasound promotes neovascularization and the expression of angiogenic factors [73]. Other studies have shown that shockwave ultrasounds act directly on the differentiation of osteoblasts, influencing the translation of genes for morphogenetic proteins and prostaglandin E2 receptors [74]. As far as the biomechanical figures are concerned, percussive ultrasound is transmitted like a sound wave through the skin. This creates compression in the bone. The result it yields is direct and indirect. Initially, it creates tensile forces that are converted into cavitation forces. This, in turn, creates hematoma formation, cell death, and bone formation (Table 4) [75].

**Table 4:** Extracorporeal shockwave therapy.

Author	Patients	Union rate (%)
Alkhawashki HM [75]	44	75%
Xu ZH et al. [76]	69	75.8%
Bara T and Synder M [77]	81	83%
Biedermann R et al.[78]	73	56%
Wang CJ et al. [79]	72	80%
Schaden W et al.[80]	115	75.7%
Rompe JD et al. [81]	43	72%
Vogel J et al. [82]	48	60.4%
Kuo SJ et al. [83]	22	63%
Valchanou VD et al.[84]	79	85.4%

The review of the literature shows that ultrasounds could have a beneficial effect on the healing of fractures [75-86]. A study compares patients with delayed unions after long bone fixation, and the authors, by the application of nails, achieved a union rate of 60%. In comparison, in another group, they chose a combination of dynamization and ultrasound, and the union rate increased to 88% [85]. The application of shockwave ultrasound to fractures is necessary to be performed in an operating room under fluoroscopic control, and patients should be required to undergo general or regional anesthesia. Generally, no adverse reactions were reported during the application of ultrasound. What is certain is that they have the disadvantage of the need for anesthesia [86]. According to a recent review study, ESWT is a time-saving procedure without adverse effects and should be considered as a first-choice treatment for tibial nonunions [87]. Furthermore, ESWT is a useful method in cases of nonunions in anatomical position and hypertrophic nonunions [88].

## Conclusions

Despite the fact that more than half a century has passed since the first report of biophysical methods, their use by the orthopedic community remains controversial. Although the studies mentioned earlier attempt to highlight the effectiveness of biophysical stimulation as a therapeutic method for fracture healing, many factors affect the outcome, such as local and systematic parameters that depend on the patient himself and those that depend on machines. More randomized studies are needed, and indeed, cooperation of many different specialties, such as doctors, physiotherapists, biologists, and industrial and technical staff in the relevant industry, to make safer and stronger conclusions.
